# A Labile Pool of IQGAP1 Disassembles Endothelial Adherens Junctions

**DOI:** 10.3390/ijms140713377

**Published:** 2013-06-27

**Authors:** Zhiguo Yuan, Wentao Zhang, Wen Tan

**Affiliations:** 1 Department of Anesthesiology, 1st Affiliated Hospital, China Medical University, Shenyang 110001, Liaoning, China; E-Mail: zhiguoyuan_73@aliyun.com; 2 School of Bioscience & Bioengineering, South China University of Technology, Guangzhou Higher Education Mega Center, Guangzhou 510006, Guangdong, China; E-Mail: wtzhang9@gmail.com; 3 Nanotides Inc., 401 Professional Drive, Suite 130, Gaithersburg, MD 20879, USA

**Keywords:** IQGAP1, Endothelial Adherens Junction, VE-cadherin, *N*-cadherin

## Abstract

Adhesion molecules are known to play an important role in endothelial activation and angiogenesis. Here we determined the functional role of IQGAP1 in the regulation of endothelial adherens junctions. VE-cadherin is found to be associated with actin filaments and thus stable, but IQGAP1 at intercellular junctions is not bound to actin filaments and thus labile. Expression of GFP labeled VE-α-catenin is shown to increase the electrical resistance across HUVEC monolayers and diminishes endogenous labile IQGAP1 at the intercellular junctions. Knockdown of endogenous IQGAP1 enhances intercellular adhesion in HUVECs by increasing the association of VE-cadherin with P120 and β-catenin. IQGAP1 knockdown also decreases the interaction of *N*-cadherin with P120 and β-catenin. Together, these results suggest that a labile pool of IQGAP1 at intercellular junctions disassembles adherens junctions and thus impairs endothelial cell-cell adhesion.

## 1. Introduction

Endothelial cell adhesion is fundamental to a number of physiological and/or pathological processes, including vascular barrier function, vascular permeability, inflammation and angiogenesis [1–3]. Endothelial cell adhesion is mediated via both tight junctions and adherens junctions. Tight junctions play an important role in the regulation of vascular barrier function and vascular permeability; while adherens junctions, containing primarily vascular endothelial (VE)-cadherin and catenins, are mainly involved in endothelial activation and angiogenesis [4]. VE-cadherin is comprised of an extracellular domain, a transmembrane region, a juxtamembrane region, and a highly conserved cytoplasmic tail [5,6]. In the presence of Ca^2+^, extracellular domains of VE-cadherin of adjacent cells bind head-to-head forming transdimers to mediate weak cell-cell adhesion. For strong adhesion, VE-cadherin trans-dimers bind to each other forming cis-dimers and clusters [4,7–9]. For cell-cell compaction, the cytoplasmic tail of VE-cadherin binds to the actin cytoskeleton through ß-catenin and a-catenin. A fourth catenin, p120, binds to the juxtamembrane region of VE-cadherin, inducing stabilization of VE-cadherin [10] and cadherin compaction [11]. Therefore, the strength of endothelial cell-cell adhesion is finely tuned by the dynamic assembly or disassembly of cadherin-catenin-actin complexes.

IQGAP1 is a scaffolding protein that binds directly to the adherens junction proteins, E-cadherin and ß-catenin, and co-localizes with these proteins at cell-cell contacts in mouse L fibroblasts expressing E-cadherin (EL cells) and in Madin Darby Canine Kidney (MDCK) epithelial cells [12]. Kaibuchi and co-workers hypothesize that the binding of IQGAP1 to ß-catenin competitively interferes with the binding of α-catenin to the E-cadherin/β-catenin complex and dissociates cell-cell adhesion, presumably by unlinking the complex from the actin cytoskeleton [12–14]. However, IQGAP1 also binds to filamentous actin and functions to cross-link and stabilize actin filaments [15,16] via its calponin homology (CH) domain at the *N*-terminus. Therefore, the exact role of IQGAP1 in the regulation of endothelial cell-cell adhesion needs to be determined.

In initial experiments, we determined by immunoprecipitation and immunofluorescence microscopy that IQGAP1 associated with adherens junction proteins like VE-cadherin. Immunofluorecence studies shows that IQGAP1 is not bound to cytoskeleton at intercellular junctions and thus labile in CSK buffer. When GFP labeled VE-α-catenin, a chimeric protein, was expressed, endogenous IQGAP1expression was diminished at the intercellular junctions, and the electrical resistance across HUVEC monolayers was increased, which suggests that labile IQGAP1 at cell-cell junctions inhibits endothelial cell-cell adhesion. To further determine the functional role of IQGAP1, we reduced the IQGAP1 expression level using RNA interference. Knockdown of IQGAP1 with a specific siRNA sequence enhances cell-cell adhesion by increasing the association of VE-cadherin with P120 and β-catenin. IQGAP1 knockdown is also shown to decrease the interaction of *N*-cadherin with P120 and β-catenin, which suggests a positive role of IQGAP1 in the initiation of endothelial activation.

## 2. Results and Discussion

### 2.1. IQGAP1 Interacts with Proteins of Adherens Junction

**Figure 1:**
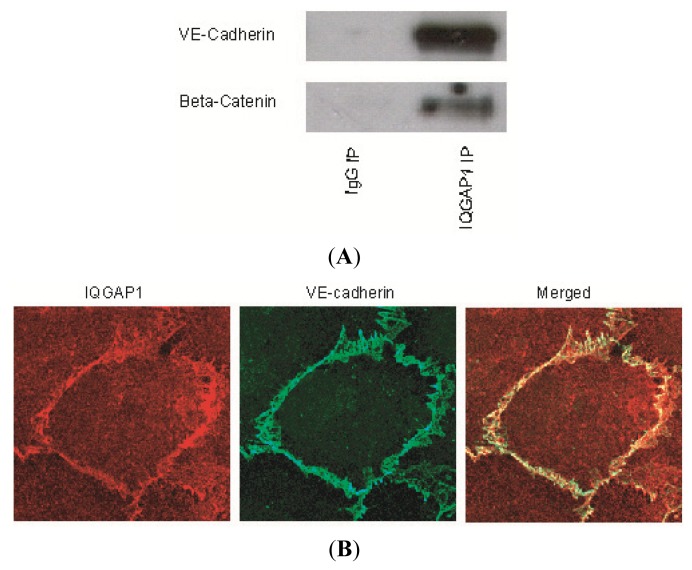

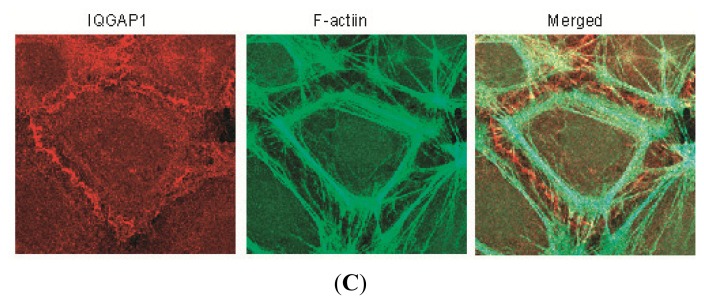
IQGAP1 associates with vascular endothelial (VE)-cadherin, β-catenins and F-actin. (**A**) HUVEC cell lysate was immunoprecipited for IQGAP1, VE-cadherin and β-catenin. Data represent three independent experimental determinations; (**B**) HUVECs were also grown to confluence on precoated glass coverslips and stained for IQGAP1 and VE-cadherin. Primary antibodies were visualized with Alexa Fluor 488-labeled goat anti rabbit IgG (green) and Alexa Fluor 594-labeled goat anti mouse IgG (red). Yellow indicates where colocalization occurs. IQGAP1 was shown to colocalize with VE-cadherin at cell-cell contact sites; (**C**) IQGAP1 was visualized at the intercellular junctions (Red) and did not colocalize with F-actin (Green, Phalloidin-488).

Using immunoprecipitation and immunofluorescence microscopy we first examined whether IQGAP1 associates with proteins of the adherens junction in HUVEC monolayers. Control cell lysates were incubated with an antibody to IQGAP1 or IgG (the latter to determine antibody specificity). Immunoprecipitates were resolved by SDS-PAGE and probed with antibodies to various adherens junction proteins. The adherens junction proteins, VE-cadherin and β- (Figure 1A), as well as γ-, α-, and p120-catenins (Figure S1), formed specific complexes with IQGAP1. Localization of IQGAP1 in confluent endothelial cell monolayers was also viewed by immunofluorescence microscopy following co-incubation with primary antibodies to IQGAP1 and VE-cadherin (Figure 1B) or one of the catenins, β-, γ-, α-, or p120- (Figure S1). IQGAP1 was localized in the cytoplasm and at cell-cell contacts (Figure 1B, IQGAP1). In the cytoplasm, IQGAP1 was evenly distributed at an intermediate level. At the intercellular junctions, IQGAP1 co-localized with VE-cadherin (Figure 1B, Merged) and the catenins at a higher level.

### 2.2. IQGAP1 Is Labile at Intercellular Junctions and Stable in the Cortex

**Figure 2:**
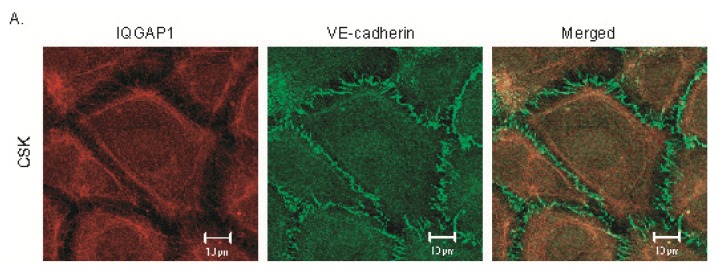

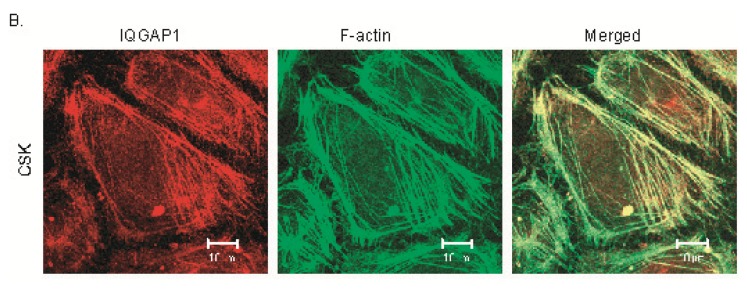
Labile IQGAP1 at intercellular junctions and Stable IQGAP1 in the cortex. HUVEC monolayers were permeabilized with Cytoskeleton buffer containing 0.5% Triton X-100 (CSK-Tx buffer) to remove labile proteins. (**A**) After CSK-Tx Buffer was added, IQGAP1 was only visualized in the cortex (Red), but not at the junctions. However, VE-cadherin was still seen at the junctions (Green); (**B**) After CSK-Tx Buffer was added, IQGAP1 was only visualized in the cortex (Red), and colocalizes with F-actin (Yellow).

Cytoskeleton buffer containing 0.5% Triton X-100 (CSK-Tx buffer), a non-ionic detergent, was used to extract labile proteins not linked to the cytoskeleton under near physiological conditions of ionic strength and pH [17]. To determine whether the junctionally-located IQGAP1 observed in Figure 1 was associated with the cytoskeleton, HUVEC monolayers were permeabilized with CSK-Tx buffer to remove labile proteins, and then fixed with 4% paraformaldehyde. In control cells, IQGAP1 and VE-cadherin were found at cell-cell contacts (Figure 1B). However, IQGAP1 disappeared from most intercellular junctions after removal of labile proteins by the permeabilizing buffer, indicating that most junctionally-located IQGAP1 is labile and not bound to the cytoskeleton (Figure 2A, IQGAP1). However, the majority of stable VE-cadherin still remained at intercellular junctions, suggesting that most junctional VE-cadherin is bound to actin filaments (Figures 1B and 2A, VE-cadherin). In contrast, stable IQGAP1 was found to bind to the cortical cytoskeleton (Figure 2B, IQGAP1) while junctional IQGAP1 was not (Figure 1C).

### 2.3. Expression of GFP Labeled VE-α-catenin Diminishes Endogenous IQGAP1 at the Intercellular Junctions

**Figure 3:**
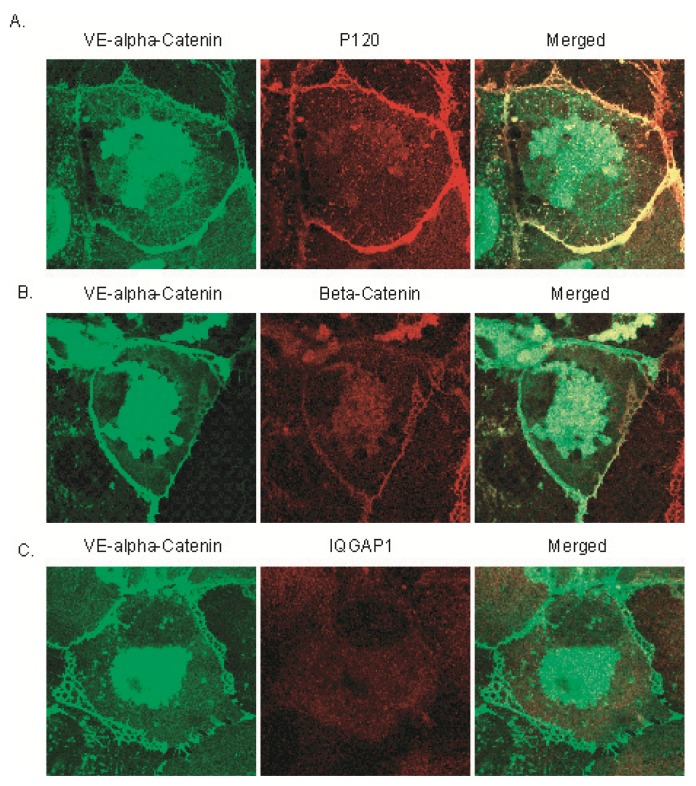
Expression of GFP labeled VE-α-catenin reduces endogenous IQGAP1 at the intercellular junctions but not β-catenin or P120. HUVECs were grown to confluence on precoated glass coverslips and stained for IQGAP1, or P120, or β-catenin (red). VE- α-catenin is labeled with GFP (green). Yellow indicates where colocalization occurs. Endogenous P120 and β-catenin were shown to colocalize with VE- α-catenin at intercellular junctions (**A**,**B**). However, the endogenous IQGAP1 at intercellular junctions was diminished (**C**).

**Figure 4:**
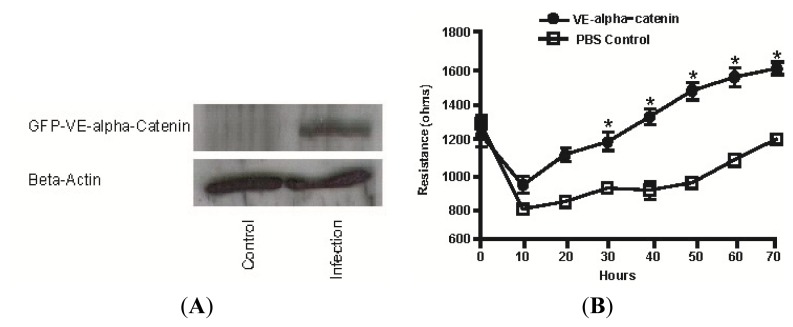
Expression of GFP labeled VE- α-catenin increases the electrical resistance across HUVEC monolayers. HUVECs were transfected with adenoviral vectors expressing GFP-labeled VE- α-catenin. (**A**) Equal amounts of protein lysates were immunoblotted for GFP or β actin. Control group was treated with PBS; (**B**) HUVECs were infected in six-well dishes for 48 h, and then trypsinized and seeded in the ECIS wells. Basal electrical resistance of HUVEC monolayer was increased by 64% from 10 to 70 h after infection (*n* = 4 for PBS treated group, *n* = 4 for GFP-VE- α-catenin expressing group).

To further study the interaction of IQGAP1 with VE-cadherin, β- and α-catenins, we infected HUVECs with adenoviral vectors expressing GFP labeled VE-α-catenin that consisted of the extracellular domain, transmembrane domain of VE-cadherin and the actin binding domain of α-catenin (without the β-catenin binding domain) (Figure 4A). Localization of IQGAP1 in HUVEC cells expressing GFP-VE-α-catenin was also viewed by immunofluorescence microscopy following co-incubation with primary antibodies to IQGAP1, β-, or p120-catenin (Figure 3). GFP labeled VE-α-catenin IQGAP1 was clearly found both in the cytoplasm and at cell-cell contacts (Figure 3, VE-α-catenin). In the cytoplasm, VE-α-catenin was aggregated around the nucleus; at the cell periphery, VE-α-catenin co-localized with endogenous p120- and β-catenins (Figure 3, Merged). Endogenous p120- and β-catenins only bind to endogenous VE-cadherin since VE-α-catenin lacks the intracellular domain of VE-cadherin. However, endogenous VE-cadherin can still form clusters with exogenous VE-α-catenin through the extracellular domain. VE-α-catenin is always connected to actin filaments and therefore overexpression of VE-α-catenin results in compaction of adherens junctions. Endogenous IQGAP1expression was diminished at the intercellular junctions (Figure 3, IQGAP1) and significantly reduced in the cytoplasm, compared with control cells (Figure 1, IQGAP1) - suggesting that endogenous IQGAP1 is excluded from clusters of endogenous VE-cadherin and exogenous VE-α-catenin. The colocalization of VE-α-catenin and p120- or β-catenins (Figure 3A,B) at intercellular junctions indicates that both p120- and β-catenins are still connected to cytoskeleton similar to endogenous VE-cadherin (Figure 2A). Thus, the clusters of VE-cadherin and catenins at intercellular junctions now consist of both exogenous VE-α-catenin and endogenous VE-cadherin-(β-, p120- and α-) catenins, but the labile pool of IQGAP1 was excluded from the compacted adherens junctions (also in Figure 2A).

### 2.4. Expression of GFP Labeled VE-α-catenin Increases the Electrical Resistance across HUVEC Monolayers

HUVEC cells were transfected (test) or treated with PBS (control) in six-well dishes for 48 h, then plated on 8w10E ECIS wells (Applied Biophysics, Troy, NY, USA) and exposed to continuous monitoring of electrical resistance for an additional 70 h. After 20 h, the electrical resistance of the HUVEC monolayers was 1100 and 850 ohms, respectively, for the VE-α-catenin adenoviral infected and PBS-treated groups (Figure 4B). This difference of ~250 ohms in electrical resistance increased to ~600 ohms at 50 h and then decreased to ~400 ohms by 70 h (*n* = 4). The maximal increase of electrical resistance in VE-α-catenin expressing HUVECs observed was 1.9 fold at 50 h (*p* < 0.01).

### 2.5. Knock-Down of IQGAP1 by siRNA Increases the Electrical Resistance across HUVEC Monolayers

**Figure 5:**
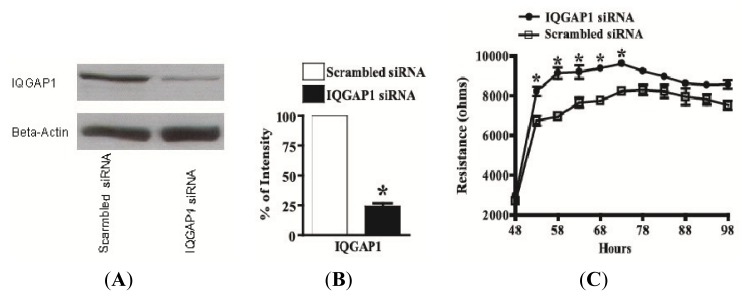
IQGAP1 knock-down increases basal resistance of HUVECs. HUVECs were transfected with 0.4 uM IQGAP1 siRNA or scrambled siRNA. (**A**) Equal amounts of protein lysate from cells transfected with scrambled siRNA and IQGAP1 siRNA were immunoblotted for IQGAP1 and β-actin; (**B**) The relative amounts of IQGAP1 in cells transfected with scrambled siRNA and IQGAP1 siRNA were quantified, and 75% IQGAP1 was knocked down by siRNA (*n* = 16); (**C**) HUVEC monolayers were transfected in six-well dishes for 48 h, then trypsinized and seeded in ECIS wells. Basal electrical resistance of HUVEC monolayers was continuously monitored by ECIS and resistance across cells transfected with IQGAP1 siRNA was higher than that with scrambled siRNA (*n* = 7). *p < 0.05 *vs.*scrambled siRNA at specific time points.

HUVECs were seeded on six-well dishes and transfected with IQGAP1 siRNA or scrambled siRNA using oligofectamine. 48 h post- transfection with IQGAP1 siRNA, the protein level of IQGAP1 was reduced by 75% (Figure 5A,B) when compared with levels of ß-actin as an internal normalization control in each lane of an immunoblot (Figure 5A). No significant cellular toxicity was detected by comparing the levels of IQGAP1 and ß-actin in HUVECs that were untreated, oligofectamine-treated, or transfected with scrambled siRNA (data not shown). Reduction of IQGAP1 by siRNA was also observed by immunofluorescence microscopy with an anti-IQGAP1 polyclonal antibody. HUVECs were transfected with IQGAP1 siRNA or scrambled siRNA in six-well dishes. After 48 h, cells were trypsinized and reseeded onto precoated glass coverslips for another 24 h. IQGAP1 siRNA, as compared with scrambled siRNA, dramatically reduced the immunofluorescent staining of IQGAP1 (data not shown).

According to the hypothesis of Kaibuchi and co-workers (26), the binding of IQGAP1 to ß-catenin loosens the adherens junction and causes cell separation. To determine the effect of reducing IQGAP1 on endothelial cell-cell adhesion, HUVECs were transfected in six-well dishes for 48 h, trypsinized, and seeded on ECIS wells for an additional 50 h. 48 h post-transfection and within five hours of seeding on the ECIS wells, electrical resistance of HUVEC monolayers were 8,200 and 6,700 ohms, respectively, for the IQGAP1 siRNA-treated and scrambled siRNA-treated groups (Figure 5C). This difference of ~1500 ohms in electrical resistance increased to ~2200 ohms at 58 h and then decreased to ~1400 ohms by 73 h post-transfection. After ECIS, cells on the active electrode were immunostained for IQGAP1 to demonstrate reduction of this protein by siRNA silencing and for VE-cadherin (to allow cell counting and to exclude any gain or loss of cells in both groups).

### 2.6. IQGAP1 Knockdown Increased Association of VE-Cadherin with p120- and β-Catenins; Opposite Response Occurred with *N*-Cadherin

**Figure 6:**
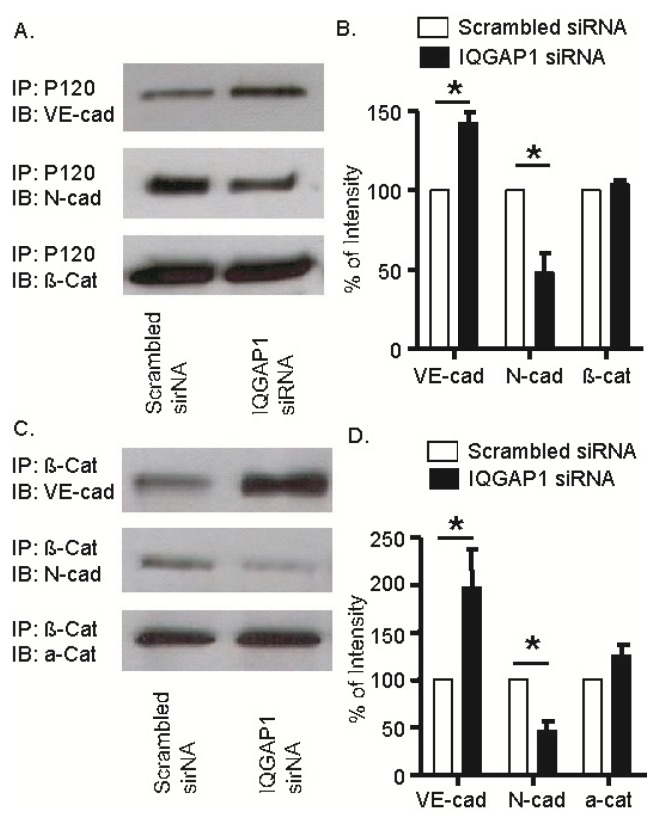
IQGAP1 knockdown increases association of VE-cadherin with p120 and β-catenin, but decreases the association of *N*-cadherin with p120 and β-catenin. HUVECs were seeded on six-well dishes for 24 h and transfected with scrambled or IQGAP1 siRNA for another 48 h. HUVEC lysates were immunoprecipitated with antibodies against p120 or β-catenin, and then blotted for VE-cadherin, *N*-cadherin, β-catenin and α-catenin. Representative immunoblots (**A**,**C**) and quantification of three independent immunoblots after normalization to β-actin (**B**,**D**) demonstrated that reduction of IQGAP1 increased the association of p120 (B) and β-catenin (D) with VE-cadherin (VE-cad) and decreased the association of p120 (B) and β-catenin (D) with *N*-cadherin (*N*-cad). Association of p120 with β-catenin (β-cat, A,B) or β-catenin with α-catenin (α-cat, C,D) did not change. * *p* < 0.05 *vs.* respective scrambled siRNA.

To understand the cellular mechanism for the higher endothelial electrical resistance in IQGAP1 siRNA-treated cells, we determined if the reduction in IQGAP1 affected the association of VE-cadherin and *N*-cadherin with the catenins. Lysates from HUVEC cells transfected with IQGAP1 siRNA or scrambled siRNA for 48 h were incubated with antibodies to p120 or β-catenin. The resultant immunoprecipitates were subjected to SDS-PAGE and probed with antibodies to VE- cadherin, *N*-cadherin, and β- and α-catenins. Association of VE-cadherin with p120 was increased by almost 50% (Figure 6A,B) and with β-catenin by ~60% (Figure 6C,D). In contrast, the association of *N*-cadherin with both p120 (Figure 6A,B) and β-catenin (Figure 6C,D) was decreased by 50%. Associations of p120 with β-catenin (Figure 6A,B) and of β-catenin with α-catenin (Figure 6C,D) were not changed by transfection with IQGAP1 siRNA.

### 2.7. Discussion

IQGAP1 binds to E-cadherin and ß-catenin, but not to α-catenin, in L cells expressing E-cadherin (EL cells) [12]. In MDCK cells, IQGAP1 co-localizes with α-catenin[18] as viewed by immunofluorescence microscopy and interacts via its carboxy terminal domain with ß-catenin and E-cadherin [12,13]. Upon binding to ß-catenin, IQGAP1 competitively interferes with the binding of α-catenin to the Ecadherin/ß-catenin complex and dissociates cell-cell contacts, presumably by unlinking the complex from the actin cytoskeleton. Furthermore, IQGAP1 in HUVEC cells was found to link VEGFR2 to VE-cadherin and thus promotes VEGF-stimulated, ROS-dependent tyrosine phosphorylation of VE-cadherin and loss of cell-cell contacts—often observed in the early stage of angiogenesis [19,20]. Together, these findings indicate that IQGAP1 interacts with proteins at adherens junctions where IQGAP1 negatively regulates cell-cell adhesion and may then promote cell migration and proliferation.

However, IQGAP1 knockdown in human microvascular endothelial cells resulted in a disruption of adherens junctions, and thus a reduction in barrier function [20]. The multifunctional roles of IQGAP1 in the regulation of endothelial barrier have to be further validated. IQGAP1 is a scaffolding protein that has multiple protein-interacting domains. Via the calponin homology (CH) domain at the *N*-terminus, IQGAP1 binds to filamentous actin and functions to cross-link and stabilize actin filaments [14–16]. Its IQ domain mediates the association of IQGAP1 with calmodulin [21], and its G-protein binding domain (GRD) at the *C*-terminus binds to activated Rac and Cdc42 [22]. IQGAP1 binds directly to the adherens junction proteins, such as E-cadherin and β-catenin, and colocalizes with these proteins at cell-cell contacts in mouse L fibroblasts expressing E-cadherin (EL cells) and in Madin Darby Canine Kidney (MDCK) epithelial cells [12].

In HUVEC cells, we found that a labile pool of IQGAP1 formed complexes with VE-cadherin and β-, γ-, α-, and p120-catenins at cell-cell contacts, and a stable pool of IQGAP1 in the cytoplasm formed complexes with cytoskeleton. To determine the exact functional role of labile IQGAP1 at intercellular junctions, we induced strong cell-cell adhesion by overexpressing VE-α-catenin in HUVEC monolayers and found that endogenous IQGAP1 is diminished at the intercellular junctions. The exogenous GFP labeled VE-α-catenin mainly reduced junctional IQGAP1, but not cytoplasmic IQGAP1, resulting in an increase in the basal electrical resistance by 64%. These findings are indirect assessments of an increased linkage of the cadherin/catenin complex to the stable cytoskeleton. In this study, we show that IQGAP1 knockdown had a positive influence on cell-cell adhesion, as assessed by the continuous measurement of electrical resistance across HUVEC monolayers. By confocal microscopy, we found that more junctional IQGAP1 was reduced by siRNA than cytosolic IQGAP1, which may be cell-density dependent. Since the integrity of endothelial barrier is often changed in physiological and pathological conditions, different endothelial cell densities may represent distinct biological processes, such as angiogenesis, inflammation and increased vascular permeability, *etc.*

## 3. Experimental Section

### 3.1. Materials

Fetal bovine serum was purchased from Atlanta Biologicals (Norcross, GA, USA), and gentamicin sulfate was from ICN Biomedicals, Inc. (Aurora, OH, USA). Newborn calf serum and bovine brain extract were from Cambrex Corporation (East Rutherford, NJ, USA). Type I collagenase was from Worthington Biochemical Corporation (Lakewood, NJ, USA). Polyclonal antibodies to VE-cadherin, IQGAP1, and p120 were from Santa Cruz Biotechnology, Inc. (Santa Cruz, CA, USA). Anti-ß-actin monoclonal and anti-a-catenin polyclonal antibodies were from Alexis Biochemicals (San Diego, CA, USA). Monoclonal antibodies directed against IQGAP1, Rac1, were from BD Biosciences (San Jose, CA, USA). Anti-IQGAP1 monoclonal (AF4) antibodies were from Upstate (Chicago, IL, USA). Gold-coated ECIS electrodes were from Applied Biophysics (Troy, NY, USA). Peroxidase conjugated goat anti-mouse IgG, peroxidase conjugated goat anti-rabbit IgG, and peroxidase conjugated rabbit anti-goat IgG antibodies were from Chemicon International, Inc. (Temecula, CA, USA). Alexa Fluor 488-labeled phalloidin, Alexa Fluor 594-labeled phalloidin, Alexa Fluor 488-labeled goat anti-rabbit IgG, and Alexa Fluor 594-labeled goat anti-mouse IgG were from Invitrogen Molecular Probes (Eugene, OR, USA). Nitrocellulose membranes and ECL (enhanced chemiluminescence) Western Blotting detection reagents were from Amersham Biosciences (Buckinghamshire, UK), and a mini-Protean electrophoresis system was from Bio-Rad Laboratories (Hercules, CA, USA). All other chemicals were from Sigma-Aldrich (St. Louis, MO, USA).

### 3.2. Cell Culture

HUVEC cells were isolated from fresh umbilical veins with 1 mg/mL of type I collagenase and serially passaged and maintained in MCDB-131 containing 20% (*v*/*v*) newborn calf serum, 5% (*v*/*v*) human serum, 7.5 μg/mL of endothelial cell growth supplement, 4.5 μg/mL of bovine brain extract, 20 μg/mL of porcine intestinal heparin, and 50 μg/mL of gentamicin sulfate. All experiments were performed on HUVEC cells passaged less than eight times.

### 3.3. Transfection

IQGAP1 siRNA was transfected into HUVECs with oligofectamine, according to manufacturer’s instructions. Briefly, HUVEC cells were seeded on 0.2% gelatin-coated six-well culture dishes or on ECIS wells and grown to 50%–80% confluence. IQGAP1 siRNA (0.27 to 0.40 μM) or scrambled siRNA was transfected into cells with oligofectamine in serum-free MCDB-131 for 4 h. Fresh HUVEC growth medium was added, and cells were placed in a humidified environment and maintained at 37 °C and 5% CO_2_ for 2–3 days.

### 3.4. Infection

HUVECs were grown to 80% confluence in full medium and then 100pfu pAd/CMV-GFP-VE-α-catenin was added to the fresh medium. Cells were placed in a humidified environment and maintained at 37 °C and 5% CO_2_ for 2–3 days.

### 3.5. Assessment of Endothelial Barrier Function by ECIS

Continuous measurement of electrical resistance across HUVEC monolayers with ECIS was used to assess changes in endothelial barrier function [23,24]. HUVEC cells (50,000–100,000 cells) were seeded onto ECIS cultureware (0.8 cm^2^/well) precoated with 0.2% gelatin. The method of transfection and measurement of electrical resistance were conducted as per manufacturer’s instructions: cells were seeded on six-well culture dishes, transfected or infected, reseeded on ECIS wells, and monitored for changes in endothelial electrical resistance.

### 3.6. Immunoprecipitation and Immunoblotting

Cells were washed twice in ice-cold phosphate buffered saline (PBS), lysed in buffer containing 30 mM 4-(2-hydroxyethyl)-1-piperazineethanesulfonic acid (HEPES, pH 7.4), 50 mM NaCl, 1% Triton X-100, 10% glycerol, 1 mM ethylene glycol-bis(2-aminoethylether)-,*N*,*N*′,*N*′-tetraacetic acid (EGTA), 1 mM sodium vanadate, 10 mM phenylmethanesulfonyl fluoride (PMSF), and 10 μg/mL of leupeptin. Samples were clarified by centrifugation at 14,000× *g* for 5 min at 4 °C, and one-tenth of the whole cell lysate was saved for immunoblotting. The remaining material from the total cell lysate was immunoprecipitated overnight at 4 °C with the indicated antibodies. Immune complexes were collected with protein A- and G-Sepharose. After centrifugation, samples were washed three times with lysis buffer. Proteins were resolved by sodium dodecyl sulfate polyacrylamide gel electrophoresis (SDS-PAGE) and transferred to Nitrocellulose membranes using a mini-Protean electrophoresis system. Polyacrylamide gels (7.5%) were used to detect IQGAP1, VE-cadherin, *N*-cadherin, the catenins, β-, γ-, α-, and p120-, and 12% gels were used to detect occludin and claudin-5. Blots were probed with indicated primary antibodies, followed by the appropriate horseradish peroxidase-conjugated secondary antibody, and developed using enhanced chemiluminescence substrate.

### 3.7. Immunocytochemistry and Confocal Microscopy

HUVEC monolayers were fixed in 4% formaldehyde in PBS for 15 min and permeabilized in PBS containing 0.18% Triton X-100 for 15 min. Fixed cells were stained with the indicated primary antibodies for 1 h and incubated with Alexa Fluor 488-labeled goat anti-rabbit IgG and/or Alexa Fluor 594-labeled goat anti-mouse IgG for 30 min. Actin filaments were fluorescently stained with Alexa Fluor 488- or 594-labeled phalloidin. Images were generated by confocal laser scanning with a Zeiss LSM 510 confocal microscope. For the same group of images, the fluorescent signals were measured at emission wavelengths of 488 and 594 nm with the same pinhole (less than 1 μm) and the same detector gain, as well as the same amplifier offset. For all of the images, multitracking was used to prevent crosstalk of signals between channels, and the bandwidth of the emission filters was narrowed to avoid bleed-through from one channel to another.

### 3.8. Statistics

All values in the text are means ± SE. Data on the quantification of immunoblots were analyzed with a single sample *t*-Test. Data on electrical resistance were analyzed with a two-way analysis of variance with repeated measures. Differences between treatments at specific time points were further analyzed with a Bonferroni post-test. Significance was set at *p* < 0.05.

## 4. Conclusions

We demonstrated in the present study in HUVECs that, (1) IQGAP1 associates with proteins comprising the endothelial adherens junction, *i.e.*, VE-cadherin and the catenins; (2) IQGAP1 is not bound to the cytoskeleton and thus is labile at intercellular junctions; (3) VE-α-catenin expression enhances endothelial cell-cell adhesion and diminishes junctional IQGAP1; (4) IQGAP1 knockdown enhances endothelial cell-cell adhesion by increasing the association of VE-cadherin with p120- and β-catenins; (5) IQGAP1 knockdown decreases the association of *N*-cadherin with p120- and β-catenins. From above, we conclude that labile IQGAP1 at intercellular junctions disrupts cell-cell adhesion by dissembling cadherin clusters and dissociating VE-cadherin from the actin cytoskeleton. Labile IQGAP1 may also promote endothelial activation by increasing the interactions of *N*-cadherin with P120 and β-catenins.

## Supplementary Information
