# Supplementary material for: A Labile Pool of IQGAP1 Disassembles Endothelial Adherens Junctions
Source: Int J Mol Sci. 2013 Jun 27;14(7):13377–90. doi: 10.3390/ijms140713377 (PMC3742192; doi:10.3390/ijms140713377)
Supplement: Supplementary file 1 [file ijms-14-13377-s001.pdf]

Supplementary Information

Figure S1. Interaction of IQGAP1 with VE-cadherin and catenins, β, α and γ. (**a–d**) HUVEC lysates were immunoprecipitated with antibodies against IQGAP1 or IgG. Representative immunoblots from three independent experiments demonstrated that IQGAP1 associates with VE-cadherin and catenins, β, α and γ; (**e–h**) Confluent HUVEC monolayers were processed for immunofluorescence microscopy. Note co-localization of IQGAP1 with VE-cadherin and β-, α- and γ-catenins (Merged). Magnification bars = 10 μm.

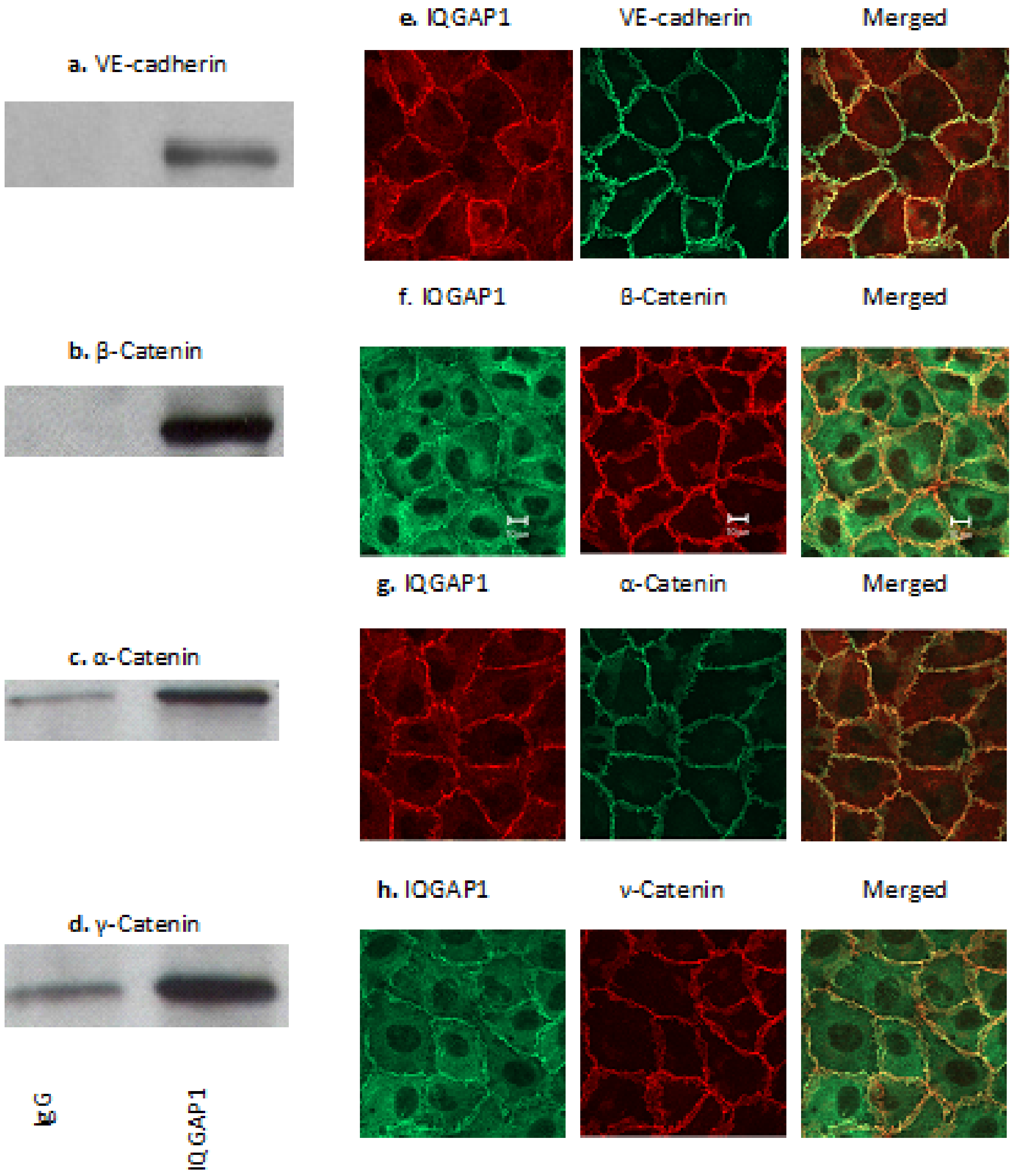

© 2013 by the authors; licensee MDPI, Basel, Switzerland. This article is an open access article distributed under the terms and conditions of the Creative Commons Attribution license (http://creativecommons.org/licenses/by/3.0/).
